# Causal Links of Type 2 Diabetes and Its Complications With Cortical Modification: A Mendelian Randomization and Mediation Analysis

**DOI:** 10.1002/brb3.71389

**Published:** 2026-05-14

**Authors:** Hong Huang, Hanyu Huang, Yanlin Gong, Tengteng Zhou, Yundan Liang, Yichao Wang

**Affiliations:** ^1^ Department of Pharmacology Dazhou Vocational and Technical College Dazhou Sichuan People's Republic of China; ^2^ Department of Orthopedic Surgery and Orthopedics, West China Hospital Sichuan University Chengdu Sichuan People's Republic of China; ^3^ Department of Pathology and Pathophysiology Chengdu Medical College Chengdu Sichuan People's Republic of China; ^4^ Division of Thyroid Surgery, Department of General Surgery, West China Hospital Sichuan University Chengdu Sichuan People's Republic of China

**Keywords:** complications, cortical modification, Mendelian randomization, mediation analysis, Type 2 diabetes

## Abstract

**Background:**

Type 2 diabetes (T2D) and its complications are linked to cognitive decline over time, accompanied by cortical abnormalities. The causality between T2D and cortical modifications, however, remains elusive.

**Methods:**

Applying two‐sample Mendelian randomization (MR), we probed causal relationships between T2D and its complications and cortical modifications. Genetic associations were elucidated via linkage disequilibrium score regression and Bayesian colocalization. An instrumental variable‐guided protein–protein interaction (PPI) network was constructed and subjected to clustering and pathway analysis. Moreover, a two‐step MR strategy was used to identify immune mediators in the causal relationship.

**Results:**

The results showed that T2D (*p* = 0.005), T2D with ophthalmic (T2D_OPTH, *p* = 0.004), or peripheral circulatory (T2D_PERIPH, *p* = 0.005) complications had a significant impact on reducing isthmus cingulate thickness. T2D_PERIPH led to an increase in the anterior cingulate surface areas, including both the caudal (*p* = 0.008) and rostral (*p* = 0.002) regions. Suggested colocalization was exclusive to T2D_OPTH and isthmus thickness, without additional genetic associations. PPI network clustering revealed causal pathway associations beyond conventional diabetes mechanisms, implicating roles in infection, addiction, and neurodegeneration. IL20RA, IgD− CD38dim B cells, HLA DR+ CD4+ T cells, and CD3 on effector memory CD4+ T cells emerged as candidate mediators of the observed causal links.

**Conclusion:**

Our study uncovers causal associations between T2D and its complications with cortical structure, highlighting the cingulate's particular vulnerability. Immuno‐metabolic dysregulation emerges as a mediator in the causal pathway connecting them, underlining inflammation control's critical importance in diabetes management.

## Graphical abstract

Mendelian randomization (MR) analyses revealed significant causal associations between T2DM (including neurological, ophthalmic, renal, and peripheral circulatory complications) and alterations in total cortical surface area, average cortical thickness, as well as region‐specific surface area and thickness across 34 cortical subdivisions.

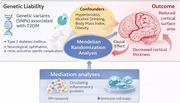

## 1 Introduction

Diabetes mellitus is a prevalent and chronic disorder worldwide, with a total number estimated to be 537 million in 2021. The prevalence of Type 2 diabetes (T2D) has increased considerably, accounting for 90% of all diabetes cases (Ahmad et al. 2022). Patients with T2D are prone to developing long‐term complications that are hard to reverse and represent the leading causes of disability and mortality (Nathan 2015). In particular, young‐onset T2D has become a widespread and distressing medical condition (Magliano et al. 2020).

Cognitive impairment is a common symptom in T2D patients, occurring at a rate of 1.5–2 times higher than the control individuals (Biessels et al. 2020). However, this impairment may be subtle in the early stages and often goes unnoticed. The impact of T2D and its complications on cognitive function has received limited attention, and the underlying mechanisms remain unclear.

Neuroimaging studies have suggested that patients with T2D exhibit structural abnormalities in the brain, which may contribute to cognitive impairment (Biessels and Despa 2018). Huang et al. (2023) found early microstructural damage in local white matter tracts in individuals with T2D. Hugenschmidt et al. (2014) reported reduced gray matter volume in the basal ganglia and concomitant decline in cognitive function in diabetic retinopathy patients. Furthermore, a multimodal magnetic resonance imaging (MRI) study with a small sample size revealed regional structural changes in the somatosensory and motor brain areas in patients with diabetic peripheral neuropathy (Selvarajah et al. 2019).

However, whether it is T2D itself or its complications that can cause changes in brain structure remains controversial. Recently, Reynolds et al. (2023) found no difference in cognitive abilities among individuals with and without T2D by employing MRI and cognitive assessments. Conversely, patients with complications showed reductions in average cortical thickness (cTH) and gray matter volume (Reynolds et al. 2023). In addition, which complications have impact on the brain structural abnormalities and which particular brain regions are involved have yet to be determined.

Mendelian randomization (MR) aims to identify genetic variants strongly associated with the exposure but independent of confounding factors, and not directly influencing the outcome (Burgess et al. 2012). These variants are then used to assess the causal effects of the exposure on the outcome. In contrast to classical epidemiological methods which grapple with challenges such as confounding factors, reverse causality, high costs, and time consumption, MR offers unique advantages as a genetic epidemiological approach (Skrivankova et al. 2021). Post MR analysis, integrating linkage disequilibrium score regression (LDSC) and Bayesian test of colocalization (COLOC) is key to enhance analytical rigor.

In our study, we first conducted a two‐sample MR analysis to investigate causal links between T2D and its complications and cortical structure. We then leveraged combined LDSC and COLOC analyses to thoroughly explore their genetic correlations. Using instrument variable (IV) loci related genes, we assembled molecular network reflecting causal connections. Moreover, mediation analysis helped uncover inflammation‐linked molecular and cellular traits intermediaries in the causal relationship observed.

## 2 Materials and Methods

### 2.1 Study Design

**FIGURE 1:**
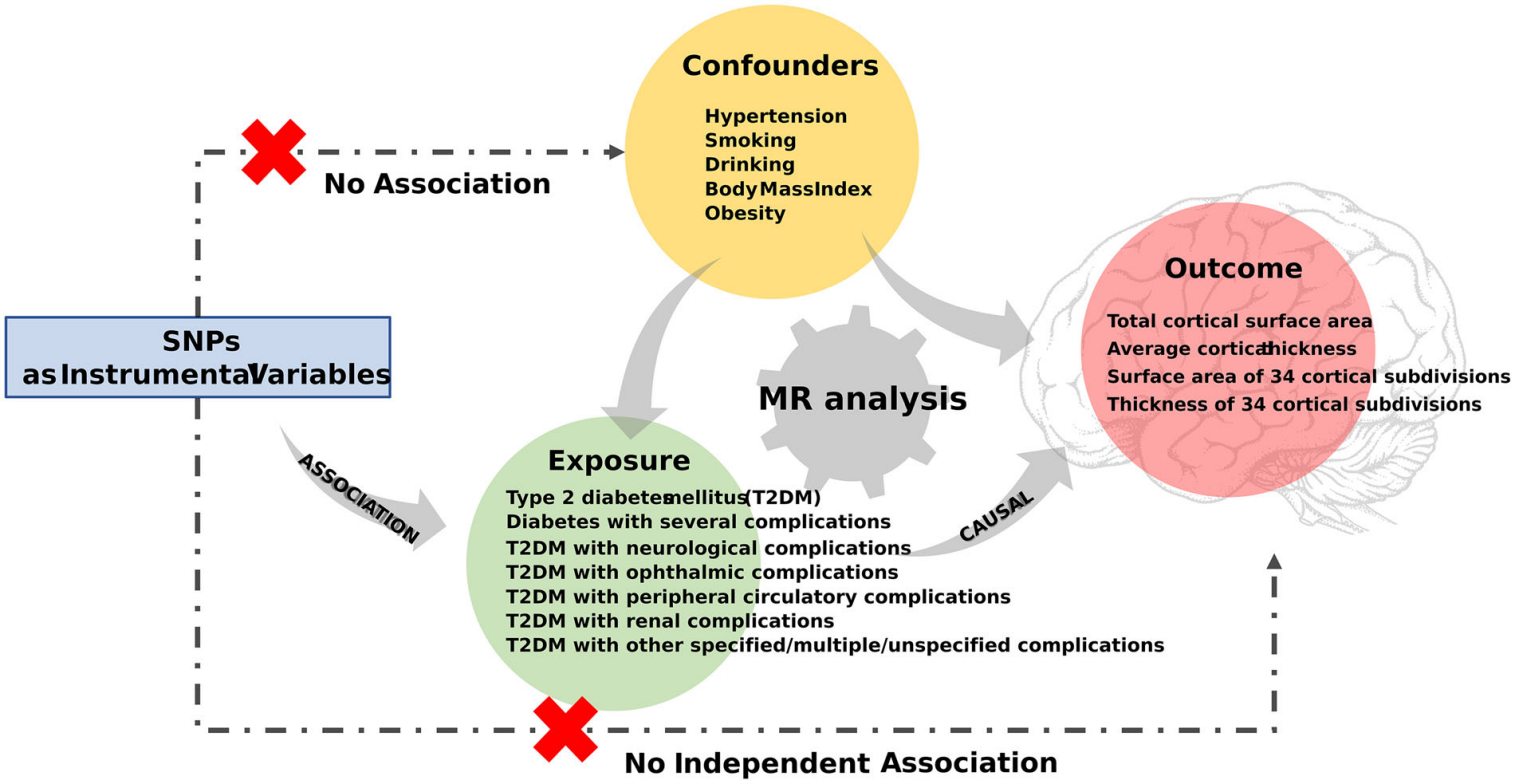
Schematic illustration of the study design.

Based on the three fundamental assumptions of MR, we first selected single nucleotide polymorphism (SNP) IVs that were strongly associated with the exposures, unrelated to confounding factors, and solely impacted the outcomes via exposures (Skrivankova et al. 2021). Subsequently, we employed five MR methods to examine the causal relationship and conducted sensitivity analyses. Figure 1 provides an overview of the study design.

### 2.2 Data Sources for T2D and Its Complications

Genetic instruments for T2D and its complications were obtained from the FinnGen research project (Kurki et al. 2023). The involved complications encompass T2D with neurological complications (T2D_NEURO), ophthalmic complications (T2D_OPTH), peripheral circulatory complications (T2D_PERIPH), renal complications (T2D_RENAL), and other complications (T2D_OTHER). Diabetes with several complications (D_SEVERAL) was also included in this study. These subjects were all clinically diagnosed and strictly classified according to hospital discharge. Table S1 displays the sample information, sources, and population characteristics of the exposure data.

### 2.3 Data Sources for Cortical Structure

The GWAS summary statistics for cerebral cortical surface area (cSA) and cTH were derived from a recent meta‐analysis performed by the ENIGMA consortium (Grasby et al. 2020). The comprehensive study was performed on 51,665 individuals from 60 cohorts, primarily of European ancestry (94%). T1‐weighted MRI was utilized for precise measurements. Our MR analysis specifically focused on individuals of European descent (*n* = 33,709). A total of 34 brain regions with specialized functions were identified in line with the Desikan–Killiany atlas (Alexander et al. 2019). The present MR analysis focused on these brain regions employing globally weighted estimates rather than relying on regional phenotypic measures. This approach mitigates the potential impact of variations in brain anatomy among individuals.

### 2.4 Selection of IVs

The IVs utilized in this study were mandated to meet and satisfy three fundamental assumptions of MR studies in order to qualify for inclusion: SNPs with genome‐wide significance (*p* < 5 × 10^−8^ for T2D and T2D_OTHER, while *p* < 5 × 10^−6^ for T2D_NEURO, T2D_OPTH, T2D_PERIPH, T2D_RENAL, and D_SEVERAL) were screened (Skrivankova et al. 2021). Given the smaller sample sizes of complication‐specific GWAS compared to overall T2D, relaxed thresholds were applied to ensure sufficient IVs. Then, a window size of 10,000 kb at a threshold of *r* ^2^ < 0.001 were settled to mitigate linkage disequilibrium (Reich et al. 2001). This process ensures that the IVs are strongly associated with exposure. Additionally, the IVs should be independent of confounding factors. PhenoScanner V2 was initially used to screen IVs for associations with potential confounders (hypertension, smoking, drinking, BMI, and obesity) (Kamat et al. 2019). Following the permanent discontinuation of PhenoScanner V2, this analysis was replicated using the IEU GWAS R package (ieugwasr)—an R interface to the IEU GWAS database integrating UK Biobank, FinnGen, and other large‐scale GWAS data. The consistency of results across both platforms confirmed the robustness of instrument selection, with no SNPs requiring exclusion based on the prespecified confounders.

Furthermore, the IVs demonstrated a clear link solely with exposure, suggesting the absence of a direct association with outcomes (Burgess et al. 2017). Palindromic SNPs, SNPs related to outcomes at a significance level of *p* < 0.05, and SNPs absent in the outcome were subsequently eliminated from the IVs. Ultimately, all SNPs displayed *F*‐statistics exceeding 10, denoting robust instrumental validity (Pierce et al. 2011).

### 2.5 MR Analysis and Sensitivity Test

Five default MR methods in the TwoSampleMR package were conducted in the analysis. The inverse variance weighted (IVW) method, being the earliest and most widely used MR method, fits a regression model without considering the intercept term and uses the inverse of the outcome variance as the weight. IVW, in our study, served as the primary method to assess potential causal relationships. However, IVW is susceptible to bias in the presence of horizontal pleiotropy. Therefore, we complemented IVW with the MR‐Egger, weighted median, simple mode, and weighted mode methods. The MR‐Egger method incorporates the intercept term primarily to evaluate the presence of horizontal pleiotropy (Burgess and Thompson 2017). The weighted median method employs the majority of genetic variants to determine causal relationships (Bowden et al. 2016). Simple mode and weighted mode are two distinct mode‐based estimate methods that exhibit robustness and accuracy in the presence of horizontal pleiotropy (Hartwig et al. 2017).

### 2.6 LDSC and COLOC Analyses

For the causally linked T2D and its complications with cortical structural changes, we subjected their GWAS raw data to both LDSC and COLOC analyses (Bulik‐Sullivan et al. 2015; Deng and Pan 2020). LDSC methods utilized regression of linkage disequilibrium scores to gauge genetic correlations (rg), with pairs exhibiting *p*‐values below 0.05 deemed significant. The COLOC analysis evaluates genetic loci for shared information with different phenotypes using five hypotheses: H0–H4 suggest no association with either trait, association with one trait but not the other, association with the second trait but not the first, two independent SNPs for both traits, and a shared SNP for both traits. A posterior probabilities of hypothesis H4 based on approximate Bayes factor (pp.H4.abf) value > 75% confirms colocalization, while > 50% indicates potential SNP sharing.

### 2.7 Protein–Protein Interaction (PPI) Network Construction, Clustering, and Functional Annotation

We extracted the nearest protein‐coding genes corresponding to the positive IVs of T2D and its complications, employing them to construct a PPI network utilizing the STRING database (Szklarczyk et al. 2023). Subsequently, these genes were clustered into three distinct groups via the k‐means algorithm, with each cluster undergoing separate Kyoto Encyclopedia of Genes and Genomes (KEGG) pathway analysis to further decipher functional enrichments and biological implications (Kanehisa et al. 2023).

### 2.8 Mediation Analysis

Mediation analysis was employed via a two‐step MR protocol (Carter et al. 2021), centering on inflammation indicators—91 circulating inflammatory proteins and 731 traits tied to immune cells (details outlined in Table S1) (Orrù et al. 2020; Zhao et al. 2023). Specifically, we conducted IVW analyses treating the relationships between T2D and its complications with inflammatory traits, and separately, between inflammatory traits and cortical structure changes, as exposure–outcome pairs. Positive results met the following criteria: (a) *p* < 0.05 from IVW, (b) no reverse causality, and (c) consistent mediator‐direction with exposure–outcome. These hits underwent delta‐method‐based SE and *p*‐value calculation, providing auxiliary references for estimating the mediation effect.

### 2.9 Statistical Analysis

We performed Bonferroni correction in the IVW analysis, with a significance threshold of *p* < 0.01 (0.05/5) as significant MR results. These results will undergo a series of sensitivity analyses. Cochran's *Q* test evaluates heterogeneity, while MR‐Egger intercept test and MR‐PRESSO global test assess horizontal pleiotropy (Rees et al. 2017; Verbanck et al. 2018). The leave‐one‐out analysis was employed to identify and exclude single SNP that has a significant impact on MR results. A threshold of *p*‐value < 0.05 was used for sensitivity analysis rejection criteria. Other statistical thresholds have been described individually within respective methodologies.

Statistical analyses were performed by the “TwoSampleMR” R package (version 4.3.1), the “ldscr” R package (version 0.1.0), and the “coloc” package (version 5.2.3).

## 3 Results

### 3.1 Causal Effects Between T2D and Its Complications and Cortical Modifications

**FIGURE 2:**
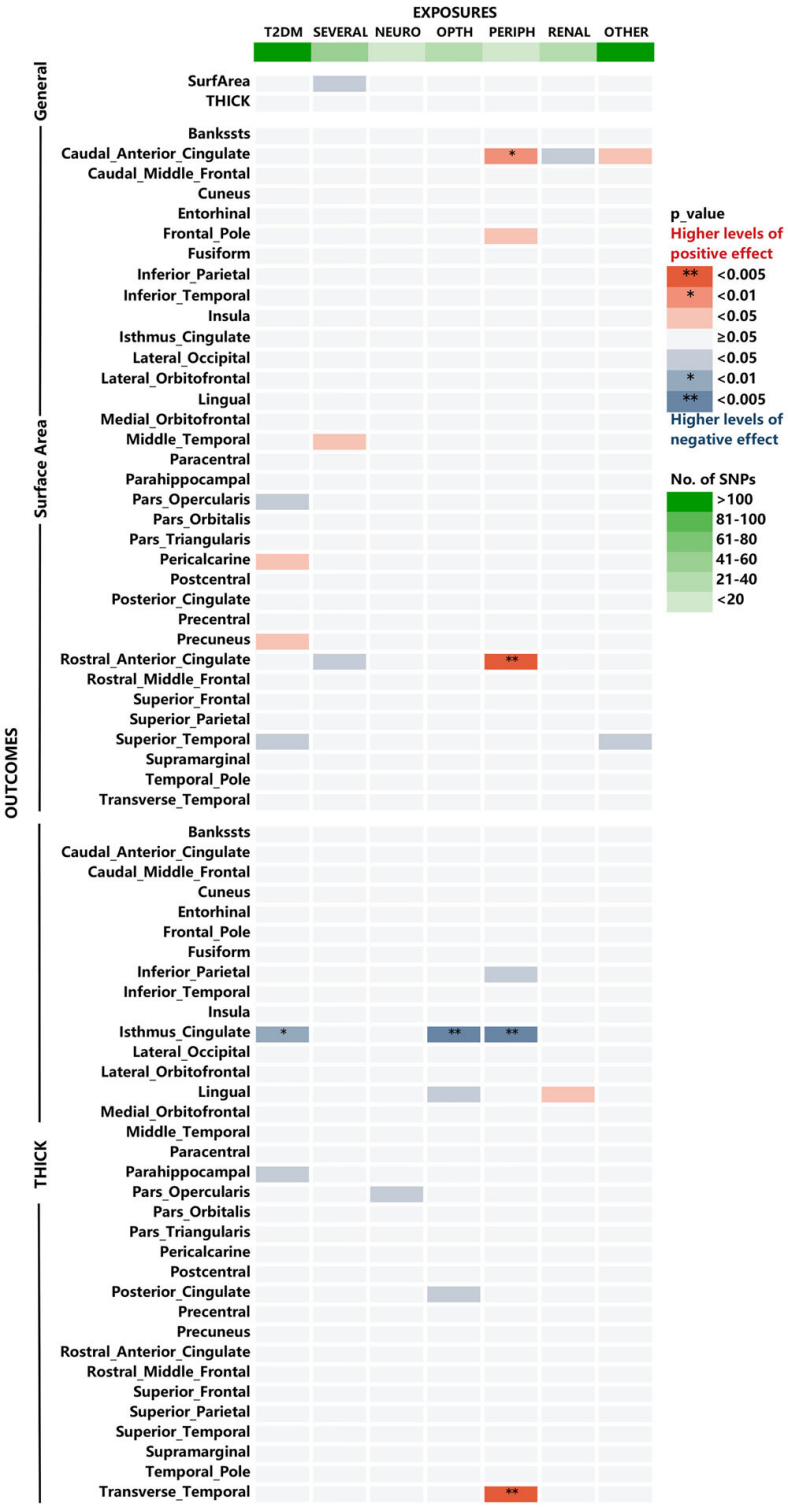
Results of IVW analyses between T2D and its complications and cortical modifications. IVW, inverse variance weighted; T2D, Type 2 diabetes.

The study investigated the causal relationship between T2D and its complications, and the average area and thickness of 35 specific cortical regions (70 traits of brain structure). Table S2 provides information of the IVs used for MR study. Results of analyses by the five MR methods are listed in Table S3. Among them, 23 showed causes with marginal statistical significance, with nine being positively correlated and 14 being negatively correlated. After Bonferroni multiple testing correction and excluding results with inconsistent beta values, five potential pairs with causal relationships were identified (Figure 2).

As shown in Table 1, T2D was found to decrease the thickness of the isthmus cingulate (beta = −0.007062 mm, 95% confidence interval [CI]: −0.009583 mm to −0.004541 mm, *p* = 0.0051). T2D with eye diseases led to a decreasing thickness in the isthmus cingulate (beta = −0.006407 mm, 95% CI: −0.008635 mm to −0.004179 mm, *p* = 0.0040). T2D with peripheral vascular diseases led to an increasing surface area in the caudal anterior cingulate (beta = 4.276 mm^2^, 95% CI: 2.672 mm^2^ to 5.88 mm^2^, *p* = 0.0077) and rostral anterior cingulate (beta = 4.781mm^2^, 95% CI: 3.219mm^2^ to 6.343mm^2^, *p* = 0.0022), while resulting in decreasing thickness in the isthmus cingulate (beta = −0.007753 mm, 95% CI: −0.010512 mm to −0.004994 mm, *p* = 0.0049). Similar results were obtained in four other methods. The scatter plots of MR analyses are shown in Figure S1.

**TABLE 1:** Causal effects between pairs of T2D and its complications and cortical structure identified through MR analysis.

Exposure	Outcome (units)	Method	No. of SNP	Beta	SE	*p*‐value
T2D	Isthmus_Cingulate_Cth	Inverse variance weighted	137	−7.06E−03	2.52E−03	0.005
	(mm)	MR‐Egger		−6.58E−03	5.86E−03	0.263
		Weighted median		−5.99E−03	4.75E−03	0.207
		Simple mode		−4.14E−03	8.85E−03	0.640
		Weighted mode		−5.22E−03	5.53E−03	0.347
T2D_OPTH	Isthmus_Cingulate_cTH	Inverse variance weighted	35	−6.41E−03	2.23E−03	0.004
	(mm)	MR‐Egger		−3.07E−03	7.58E−03	0.688
		Weighted median		−6.33E−03	3.21E−03	0.048
		Simple mode		−6.94E−03	5.42E−03	0.208
		Weighted mode		−5.95E−03	4.47E−03	0.192
T2D_PERIPH	Isthmus_Cingulate_cTH	Inverse variance weighted	11	−7.75E−03	2.76E−03	0.005
	(mm)	MR‐Egger		−8.26E−03	7.39E−03	0.293
		Weighted median		−6.20E−03	3.79E−03	0.101
		Simple mode		−1.15E−02	5.75E−03	0.073
		Weighted mode		−7.64E−03	4.73E−03	0.137
T2D_PERIPH	Caudal_Anterior_Cingulate_cSA	Inverse variance weighted	11	4.276	1.604	0.008
	(mm^2^)	MR‐Egger		5.311	4.452	0.263
		Weighted median		1.651	2.231	0.459
		Simple mode		1.385	3.760	0.720
		Weighted mode		1.455	2.720	0.604
T2D_PERIPH	Rostral_Anterior_Cingulate_cSA	Inverse variance weighted	11	4.781	1.562	0.002
	(mm^2^)	MR‐Egger		4.393	4.371	0.341
		Weighted median		3.582	2.204	0.104
		Simple mode		4.441	3.514	0.235
		Weighted mode		2.032	2.925	0.503

Abbreviations: cSA, cortical surface area; cTH, cortical thickness; MR, Mendelian randomization; SE, standard error; SNP, single nucleotide polymorphism; T2D, Type 2 diabetes; T2D_OPTH, T2D with ophthalmic; T2D_PERIPH, T2D with peripheral circulatory complications.

### 3.2 Sensitivity Analyses

We further performed sensitivity analyses on the above results to ensure their reliability (Table 2). All *p*‐values of the Cochran *Q*‐tests were above 0.05, indicating no significant heterogeneity. Both MR‐Egger intercept tests and the MR‐PRESSO global tests of all association pairs were above 0.05, suggesting the absence of significant pleiotropy. Additionally, the leave‐one‐out analysis suggested that the exclusion of any individual SNP did not lead to substantial alterations in the results (Figures S2–S6).

**TABLE 2:** Heterogeneity and pleiotropy evaluation for sensitivity test.

Exposure	Outcome	Method	Cochran's *Q* test			MR‐Egger intercept test			MR‐PRESSO global test	
			*Q*	*Q*_df	*Q*_pval	Intercept	SE	*p*‐value	RSS obs	*p*‐value
T2DM	THICK_Isthmus_Cingulate	Inverse variance weighted	148.90	135.00	0.20	−3.59E−05	3.91E−04	0.93	151.08	0.20
		MR‐Egger	148.91	136.00	0.21					
T2DM_OPTH	THICK_Isthmus_Cingulate	Inverse variance weighted	36.30	33.00	0.32	−5.08E−04	1.10E−03	0.65	38.23	0.37
		MR‐Egger	36.53	34.00	0.35					
T2DM_PERIPH	SurfArea_Caudal_Anterior_Cingulate	Inverse variance weighted	9.72	9.00	0.37	−2.57E−01	1.03E+00	0.81	11.82	0.51
		MR‐Egger	9.79	10.00	0.46					
T2DM_PERIPH	SurfArea_Rostral_Anterior_Cingulate	Inverse variance weighted	9.95	9.00	0.35	9.66E−02	1.01E+00	0.93	12.84	0.43
		MR‐Egger	9.96	10.00	0.44					
T2DM_PERIPH	THICK_Isthmus_Cingulate	Inverse variance weighted	6.75	9.00	0.66	1.26E−04	1.70E−03	0.94	7.91	0.77
		MR‐Egger	6.76	10.00	0.75					

Abbreviations: MR, Mendelian randomization; SE, standard error; T2D, Type 2 diabetes; T2D_OPTH, T2D with ophthalmic; T2D_PERIPH, T2D with peripheral circulatory complications.

### 3.3 Genetic Correlations Between T2D and Its Complications and Cortical Alterations

As depicted in Table S4, the LDSC and COLOC analyses elucidated genetic correlations between T2D and its complications and cortical alterations, with LDSC indicating no significant genetic correlation between them. Specifically, T2D demonstrated a positive association with the isthmus cingulate thickness (rg = 0.00613, *p* = 0.72). T2D_OPTH showed a negative correlation with the isthmus cingulate thickness (rg = −0.00832, *p* = 0.76), with COLOC analysis suggesting a potential shared SNP involvement as indicated by pp.H4.abf reaching 60%. T2D_PERIPH negatively correlates with the surface area of caudal and rostral anterior cingulate (rg = −0.0853 and −0.0452, respectively; *p* = 0.11 and 0.34), and positively with the isthmus cingulate thickness (rg = 0.0630, *p* = 0.16). Moreover, the other COLOC analyses implied no significant shared genetic overlap (Figure S7).

### 3.4 PPI Network Construction and Cluster‐Based Annotation

One hundred and twenty nearest genes were identified in the PPI network using IVs from T2D, T2D_OPTH, and T2D_PERIPH. Through k‐means clustering, three groups were defined with 86, 24, and 10 genes each (Table S5). KEGG pathway analysis revealed that Cluster 1 was primarily associated with pathways related to T2D, inflammation, and lipid metabolism; Cluster 2 was linked to various neurodegenerative diseases, signaling pathways, and substance addiction; and Cluster 3 was implicated in metabolic processes, O‐glycosylation, vesicle transport, and endocytosis (Figure S8 and Table S5 for details).

### 3.5 Mediation Analyses

**FIGURE 3:**
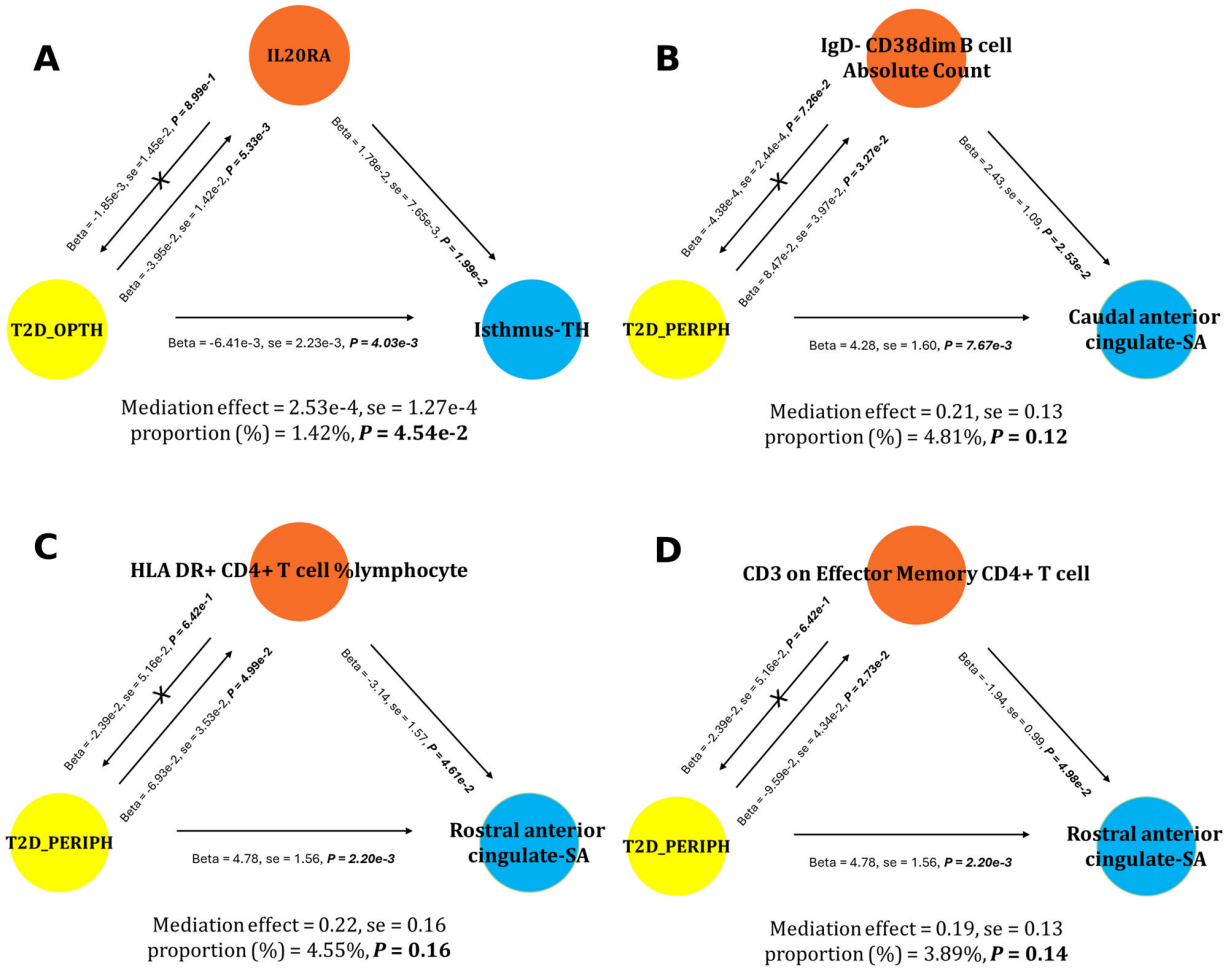
Inflammation‐mediated causal links between T2D and its complications and cingulate alterations: (A) IL20RA in T2D_OPTH‐related isthmus cingulate thinning; (B) IgD− CD38dim B cells in T2D_PERIPH affecting caudal anterior cingulate surface area; and (C, D) HLA DR+ CD4+ T cells and effector memory CD4+ T cells in T2D_PERIPH's impact on rostral anterior cingulate surface area. HLA, human leukocyte antigen; IgD, immunoglobulin D; IL20RA, interleukin‐20 receptor subunit alpha; T2D, Type 2 diabetes; T2D_OPTH, Type 2 diabetes with ophthalmic complications; T2D_PERIPH, Type 2 diabetes with peripheral circulatory complications.

In search of mediators among inflammatory cytokines and immune cell traits, we found that IL20RA potentially mediated the causal link between T2D_OPTH and changes in isthmus cingulate thickness (mediation effect = 2.53e−4, SE = 1.27e−4, proportion = 1.42%, *p* = 4.54e−2). IgD− CD38dim B cell absolute count may mediate the causality between T2D_PERIPH and the change in caudal anterior cingulate surface area (mediation effect = 0.21, SE = 0.13, proportion = 4.81%, *p* = 0.12). HLA DR+ CD4+ T cell percentage within lymphocytes and CD3 on effector memory CD4+ T cell were individually suggested to mediate the causal association of T2D_PERIPH with modifications in the rostral anterior cingulate surface area (mediation effects = 0.22, SE = 0.16, proportion = 4.55%, *p* = 0.16; and mediation effects = 0.19, SE = 0.13, proportion = 3.89%, *p* = 0.14, respectively). Detailed results are shown in Figure 3, and IVW analysis results are presented in Tables S6–S9.

## 4 Discussion

In this study, we investigated the causal relationships between T2D and its complications and structural alterations of the cingulate cortex using MR analyses. We found that T2D, particularly with ophthalmic or peripheral circulatory complications, was associated with reduced isthmus cingulate thickness, while peripheral circulatory complications were additionally linked to increased anterior cingulate surface area. Colocalization and pathway analyses suggested involvement of specific genetic signals and immune‐related factors, indicating that T2D and its complications may drive region‐specific cingulate cortex remodeling through these mechanisms.

Previous studies have consistently reported associations between T2D and global or regional cortical alterations (Cao et al. 2024; Chen et al. 2015; Motaghi et al. 2024). However, the extent to which these changes reflect causal effects of diabetes itself versus its complications has remained unclear. With the application of the Bonferroni method to correct for multiple test comparisons, our MR analysis revealed that T2D did not have a causal relationship with the surface area and thickness of most brain regions, except for causing thinning of the isthmus cingulate. In contrast, the complications of T2D, especially T2D_PERIPH, had an effect on the surface area and thickness of several cortical regions. These findings extend previous observational neuroimaging studies by providing genetically informed evidence that complication burden, rather than T2D diagnosis alone, are primary drivers of diabetes‐related cortical vulnerability (Reynolds et al. 2023).

Among the significant results, the exposure of T2D_PERIPH emerged as the most common factor. According to the ICD‐10 classification, T2D_PERIPH includes diabetic gangrene, peripheral angiopathy, and ulcer. When these complications occur, individuals are more likely to exhibit thickness reduction of the isthmus cingulate. Persistent hyperglycemia induces oxidative stress and inflammation, leading to endothelial dysfunction and reduced local cerebral blood flow, which may be the main cause of structural changes (Gorst et al. 2015; Y. Li et al. 2023; Narula et al. 2018). Moreover, T2D_OPTH, including cataract and retinal disease, also lead to reduced thickness of the isthmus cingulate. The pathogenesis of diabetic cataract lies on chronic oxidative damage and abnormal glucose metabolism leading to the accumulation of harmful byproducts (Mrugacz et al. 2023), while diabetic retinopathy is caused by vascular damage (H. Li et al. 2023). These findings indicate the necessity of integrated management of blood glucose, oxidative stress, and vascular health in the prevention and treatment of diabetic eye disease.

The outcome of isthmus cingulate was the most frequently observed in the significant results. As a limbic structure bridging the posterior cingulate cortex to the hippocampus, it is integral to spatial memory, emotion regulation, and social cognition (Apps et al. 2016; Saygi et al. 2023; van Heukelum et al. 2020). Isthmus cingulate has a high density of neurons and complex neural network, which ensure its efficient information processing ability (Hau et al. 2019). However, it is more susceptible to ischemia and hypoxia. Abnormalities in the isthmus cingulate have also been observed in studies of traumatic brain injury (Santhanam et al. 2019) and neurodegenerative diseases (Cieri et al. 2022), which are commonly associated with long‐term cognitive impairments. Moreover, T2D patients frequently experience comorbidity with mental disorders, including anxiety and depression, which may also be linked to changes in the isthmus cingulate (Carnevali et al. 2019).

Vascular complications showed a causal connection with diffuse anterior cingulate cortex (ACC) surface expansion, affecting both its caudal and rostral parts. The ACC is located in the frontal lobe of the brain between the cingulate sulcus and corpus callosum. The ACC plays a crucial role in fine motor coordination and is closely linked to reward processing, decision‐making, and emotion (Clairis and Lopez‐Persem 2023). Increased cSA has positive effects, possibly due to the fact that in T2D patients with less neuroplasticity, functional compensation can only be achieved through structural changes. Understanding these morphological changes in the ACC may provide critical insights for developing strategies to mitigate T2D‐associated cognitive decline.

No significant genetic correlations were detected by LDSC analyses for exposure–outcome pairs with positive MR results. While IVs in MR analysis effectively capture causal links between exposure and outcome, LDSC's use of the entire spectrum of genetic variants may incorporate pleiotropic or weak effects, diluting signals and yielding nonsignificant findings. In COLOC analysis, a pp.H4.abf above 50% for T2D_OPTH and isthmus cingulate thickness implicated shared genetic drivers, notably clustering near HLA genes. The top variant associated with T2D_OPTH, rs58327373, resides between HLA‐DQB2 and HLA‐DOB, while rs601148, the most significant for isthmus cingulate thickness, is situated between HLA‐DRB1 and HLA‐DQA1. Known for ties to diabetes, especially via DQ and DR region alleles affecting autoimmunity, HLA genes, though less studied in neural context, may impact brain structure through immune modulation (Zhao et al. 2024).

Following clustering of the IVs‐related genes‐based PPI network, we obtained three gene subclasses. Pathway analysis of these clusters, beyond implicating common pathways in diabetes, glucose and lipid metabolism, and cellular signaling, also revealed associations with infection processes (human T‐cell leukemia virus 1 and human papillomavirus), substance addiction (cocaine and amphetamine), and neurodegenerative diseases (Alzheimer's disease and Huntington's disease). These findings corroborate the association between T2D and its complications with brain structure and function, and furthermore, they imply that immune‐related factors may underpin the causal links between them.

We have pinpointed four immune mediators implicated in the connection between T2D and its complications, and cortical anomalies. IL20RA, a key immunomodulator, activates the JAK1‐STAT3 pathway, controlling inflammation by fine‐tuning cytokines crucial for both tumorigenesis and autoimmunity, such as IL‐10, IL‐12, and IL‐13 (Caparros and Frances 2018; Lamichhane et al. 2021). IgD− CD38dim B cells, a distinct B cell subset, while understudied, align with autoimmune implications seen in Type 1 diabetes, potentially contributing to brain inflammation (Bass and Bonami 2024; Powers 2021). HLA DR+ CD4+ T cells are central to self‐antigen presentation and autoimmune pancreatic β‐cell targeting in Type 1 diabetes. CD3‐bearing effector memory CD4+ T cells, implicated in subset dynamics during diabetic nephropathy, are integral to autoimmune pathways (Xie et al. 2024). These agents suggest that chronic autoimmune imbalance in T2D may be a source of continuous CNS inflammation that drives cortical changes.

From a translational perspective, these findings imply that neurocognitive risk stratification in T2D should incorporate complication status and immune‐inflammatory profiles rather than relying solely on glycemic indices. Early identification and aggressive management of vascular and inflammatory complications may offer a viable strategy to mitigate long‐term brain structural damage and cognitive decline.

This study also generates several testable hypotheses for future research. First, HLA‐driven immune susceptibility may prime limbic regions, particularly the isthmus cingulate for inflammation‐induced structural remodeling. Second, peripheral immune activation may propagate central neuroinflammatory cascades through blood–brain barrier dysfunction. Third, ACC surface expansion may represent a transient compensatory phase preceding decompensatory cortical thinning. Further studies integrating immune profiling, neuroimaging, and functional assessments will be essential to validate these mechanisms.

This study still has several limitations. First, the study cohort was limited to individuals of European ancestry, and the findings may not be directly generalizable to Asian populations. Nevertheless, the observed associations between T2D‐related traits and cingulate cortex structural alterations, along with potential immune‐related mechanisms, highlight pathways that may be relevant in Asian populations. Future studies using large‐scale Asian genetic datasets are needed to validate these findings. Second, there are not enough GWAS data on T2D complications with clear diagnosis and sufficient population size. Exposure data in our study has a limited number of cases compared to controls. These factors led to a relatively weak power for the IVs in some pairs. Increasing the number of samples in the future will enhance result credibility. Furthermore, we solely controlled for established major confounders, while unable to account for all conceivable confounders. This serves as a reminder that prudence is warranted when employing MR to elucidate causality.

In summary, our study highlights that T2D and its complications contribute to cingulate cortex alterations, with genetic and immune factors suggesting a key role for immune‐metabolic interactions in this process.

## Author Contributions

**Hong Huang**: methodology, writing – original draft. **Hanyu Huang**: methodology, writing – original draft. **Yanglin Gong**: methodology, writing – original draft. **Tengteng Zhou**: methodology, writing – original draft. **Yundan Liang**: conceptualization, writing – review and editing. **Yichao Wang**: conceptualization, writing – review and editing.

## Funding

This work was supported by grants from the Sichuan Research Center of Applied Psychology, Key Research Base of Philosophy and Social Sciences of Sichuan Province (no: CSXL‐24202), Sichuan Clinical Research Center for Geriatrics (no: 24 LHLNYX1‐04), Development and Regeneration Key Laboratory of Sichuan Province (no: 24 LHFYSZ1‐25), and Chengdu Medical College Research Development Fund (no: CYZZD25‐10).

## Ethics Statement

Ethics approval was not required for this study as the data were obtained from publicly available databases with unrestricted reuse permitted under an open license. The data are de‐identified and publicly accessible.

## Conflicts of Interest

The authors declare no conflicts of interest.

## Supporting information




**Supplementary Figure S1**: brb371389‐sup‐0001‐FigureS1.jpg

**Supplementary Figure S2**: brb371389‐sup‐0002‐FigureS2.jpg

**Supplementary Figure S3**: brb371389‐sup‐0003‐FigureS3.jpg

**Supplementary Figure S4**: brb371389‐sup‐0004‐FigureS4.jpeg

**Supplementary Figure S5**: brb371389‐sup‐0005‐FigureS5.jpg

**Supplementary Figure S6**: brb371389‐sup‐0006‐FigureS6.jpg

**Supplementary Figure S7**: brb371389‐sup‐0007‐FigureS7.jpg

**Supplementary Figure S8**: brb371389‐sup‐0008‐FigureS8.jpg

**Supplementary Table S1**: brb371389‐sup‐0009‐TableS1.xlsx

**Supplementary Table S2**: brb371389‐sup‐0010‐TableS2.xlsx

**Supplementary Table S3**: brb371389‐sup‐0011‐TableS3.xlsx

**Supplementary Table S4**: brb371389‐sup‐0012‐TableS4.xlsx

**Supplementary Table S5**: brb371389‐sup‐0013‐TableS5.xlsx

**Supplementary Table S6**: brb371389‐sup‐0014‐TableS6.xlsx

**Supplementary Table S7**: brb371389‐sup‐0015‐TableS7.xlsx

**Supplementary Table S8**: brb371389‐sup‐0016‐TableS8.xlsx

**Supplementary Table S9**: brb371389‐sup‐0017‐TableS9.xlsx

## Data Availability

Summary statistics data of FinnGen project can be accessed through the Fingenious services at: https://site.fingenious.fi/en/. The cortical structural data of this study are not openly available and are available from the corresponding author upon reasonable request. Data are located in controlled access data storage at ENIGMA consortium (https://enigma.ini.usc.edu/). Data on circulating inflammatory factors and immune cell traits can be found in the GWAS catalog at: https://www.ebi.ac.uk/gwas/.
