# Supplementary figures and images for: Causal Links of Type 2 Diabetes and Its Complications With Cortical Modification: A Mendelian Randomization and Mediation Analysis
Source: Brain Behav. 2026 May 14;16(5):e71389. doi: 10.1002/brb3.71389 (PMC13176085; doi:10.1002/brb3.71389)

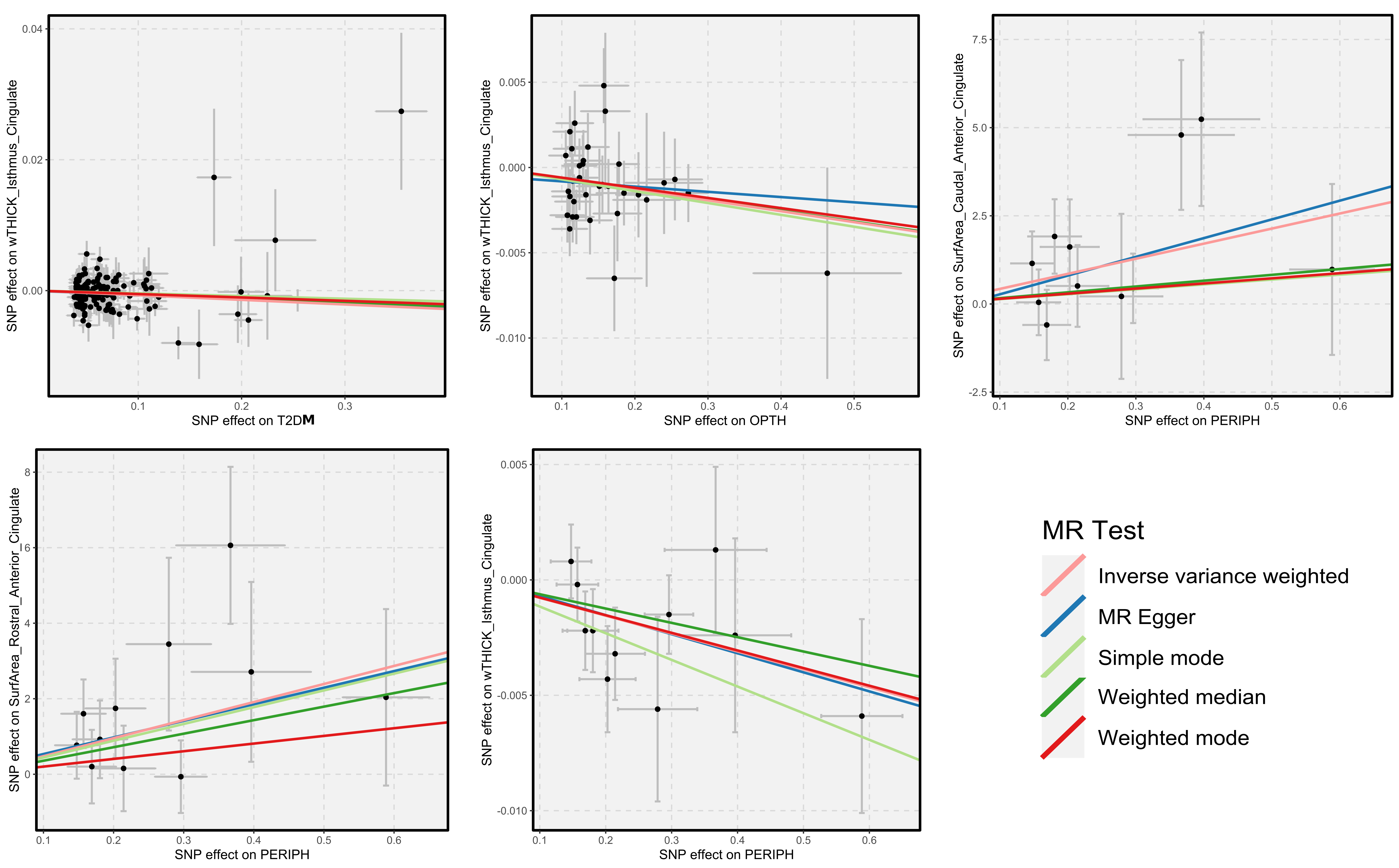

Supplement: Supplementary file 1 — Supplementary Figure S1: brb371389‐sup‐0001‐FigureS1.jpg [file BRB3-16-e71389-s011.jpg]

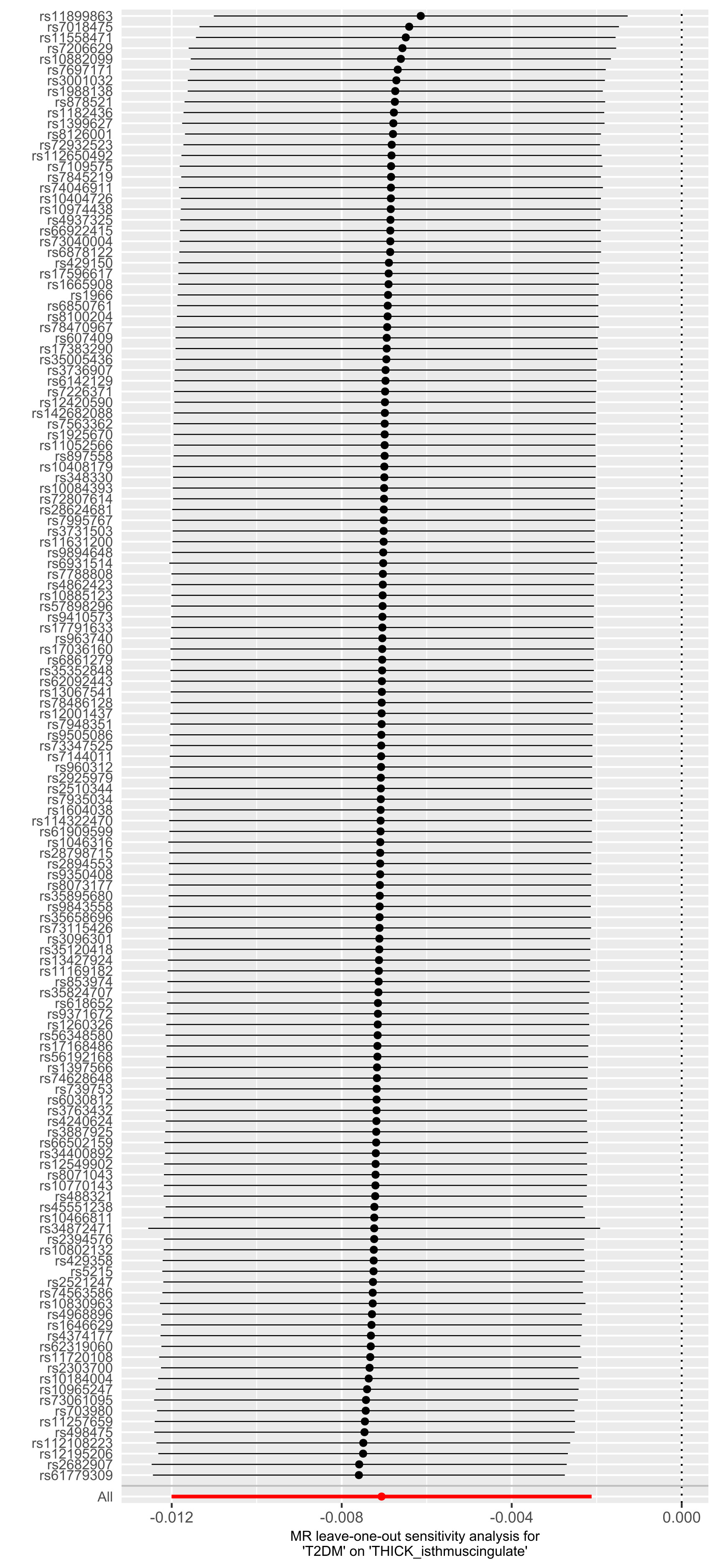

Supplement: Supplementary file 2 — Supplementary Figure S2: brb371389‐sup‐0002‐FigureS2.jpg [file BRB3-16-e71389-s010.jpg]

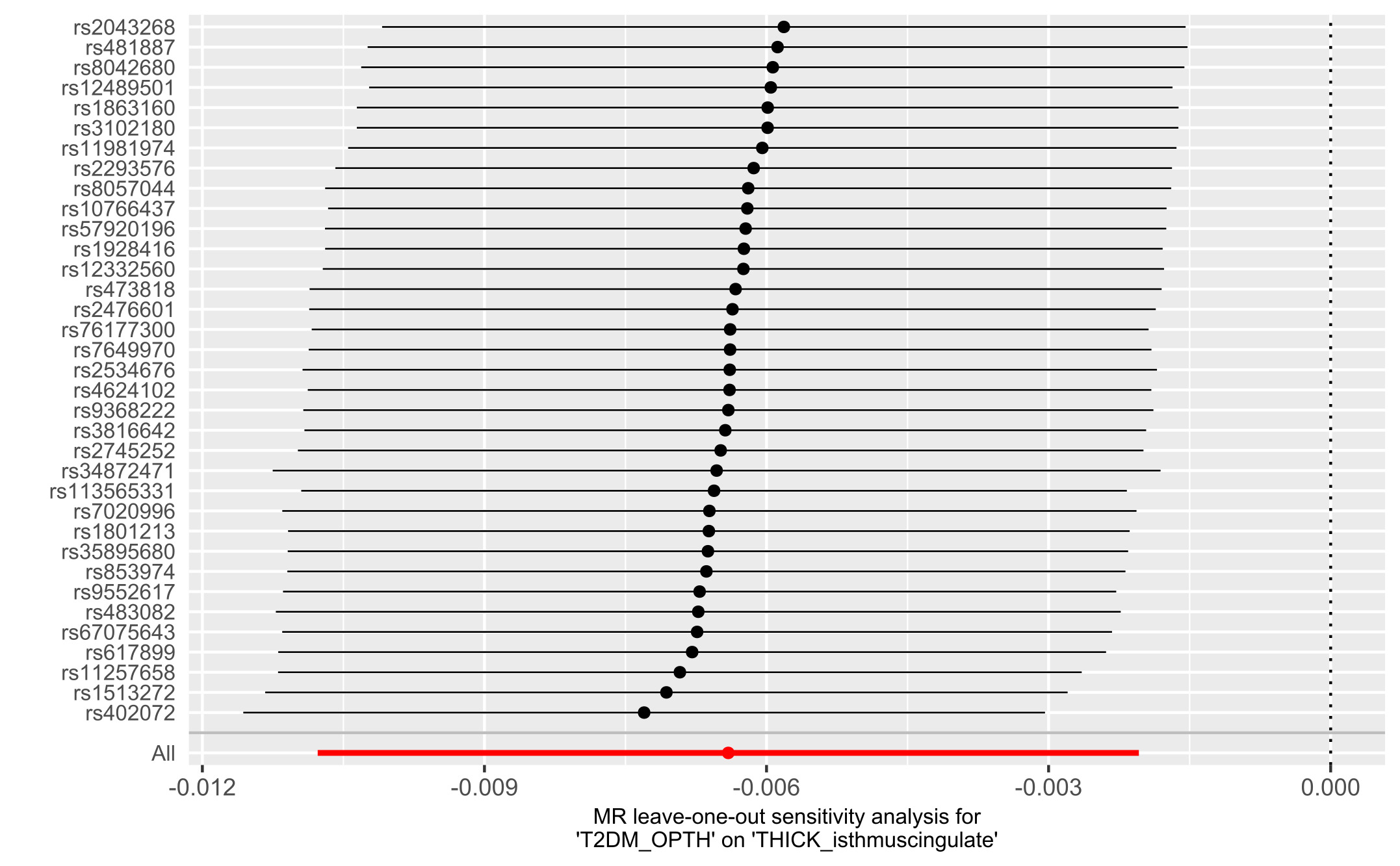

Supplement: Supplementary file 3 — Supplementary Figure S3: brb371389‐sup‐0003‐FigureS3.jpg [file BRB3-16-e71389-s003.jpg]

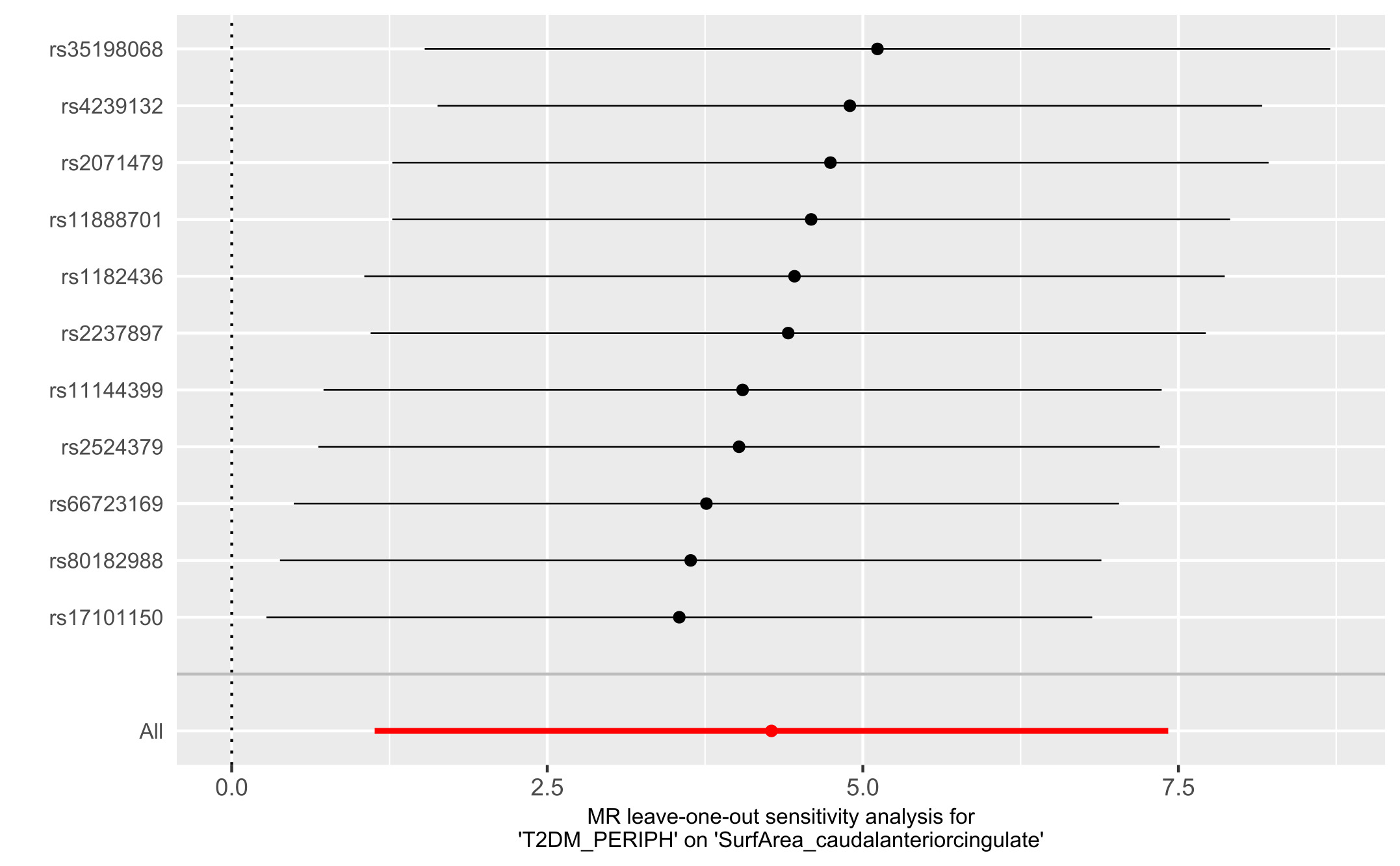

Supplement: Supplementary file 4 — Supplementary Figure S4: brb371389‐sup‐0004‐FigureS4.jpeg [file BRB3-16-e71389-s007.jpeg]

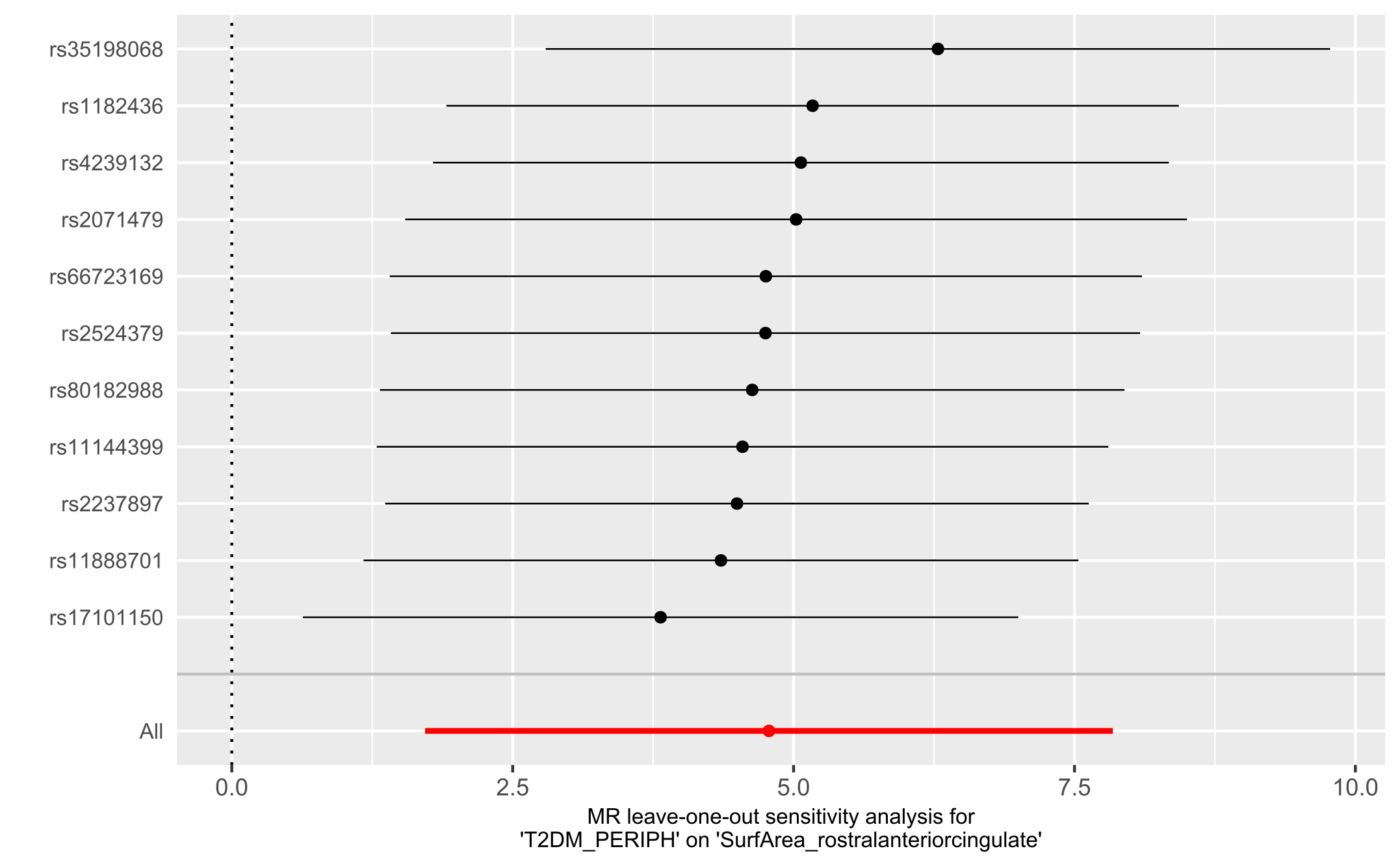

Supplement: Supplementary file 5 — Supplementary Figure S5: brb371389‐sup‐0005‐FigureS5.jpg [file BRB3-16-e71389-s004.jpg]

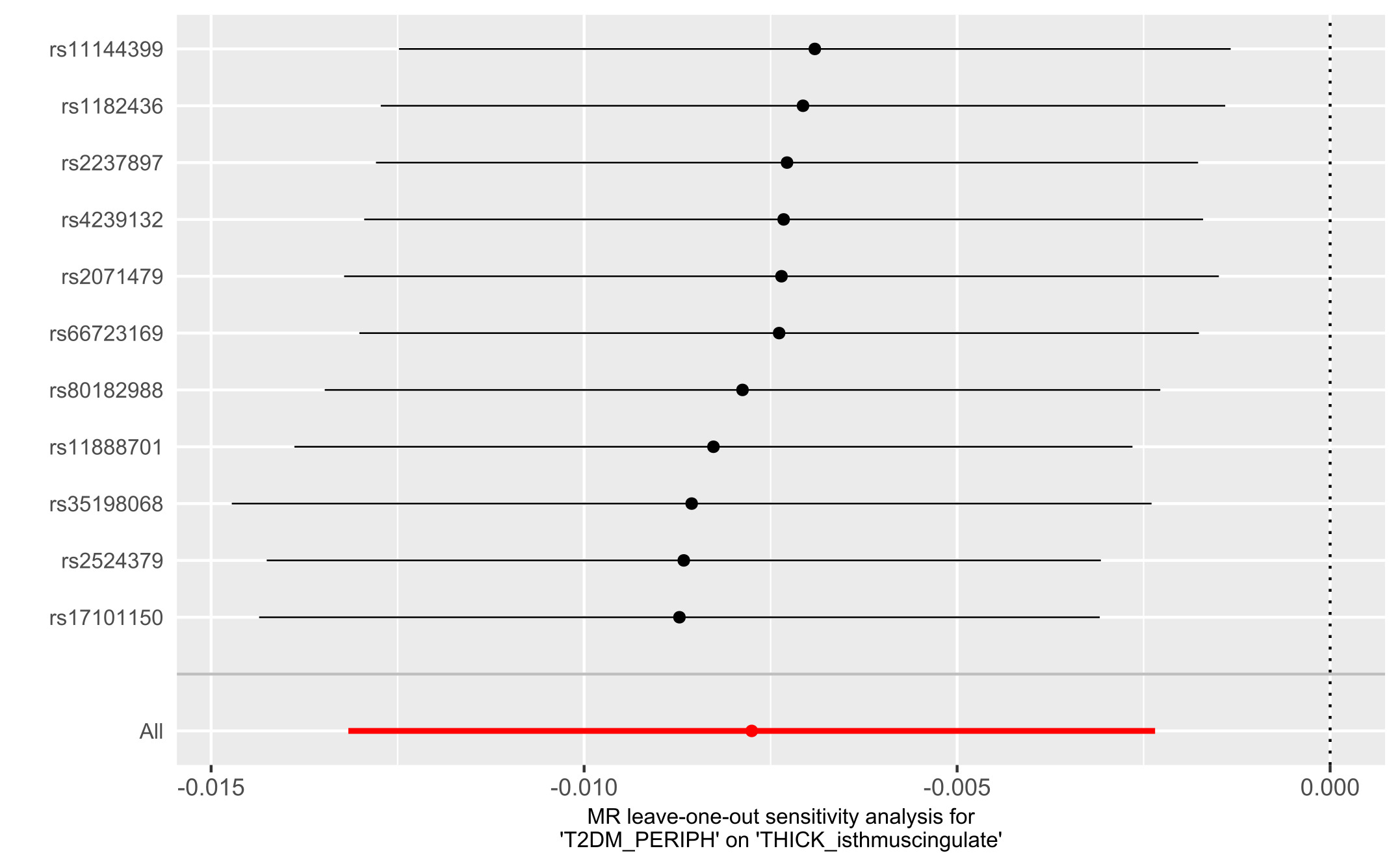

Supplement: Supplementary file 6 — Supplementary Figure S6: brb371389‐sup‐0006‐FigureS6.jpg [file BRB3-16-e71389-s006.jpg]
